# Impact of the European General Data Protection Regulation (GDPR) on Health Data Management in a European Union Candidate Country: A Case Study of Serbia

**DOI:** 10.2196/14604

**Published:** 2020-04-17

**Authors:** Branko Marovic, Vasa Curcin

**Affiliations:** 1 Computer Centre University of Belgrade Belgrade Serbia; 2 School of Population Health and Environmental Sciences King's College London London United Kingdom

**Keywords:** privacy act, patient data privacy, data sharing, information disclosure, ethical issues, medical tourists, health care systems, public policy, policy compliance, legal aspects, international aspects

## Abstract

As of May 2018, all relevant institutions within member countries of the European Economic Area are required to comply with the European General Data Protection Regulation (GDPR) or face significant fines. This regulation has also had a notable effect on the European Union (EU) candidate countries, which are undergoing the process of harmonizing their legislature with the EU as part of the accession process. The Republic of Serbia is an example of such a candidate country, and its 2018 Personal Data Protection Act mirrors the majority of provisions in the GDPR. This paper presents the impact of the GDPR on health data management and Serbia’s capability to conduct international health data research projects. Data protection incidents reported in Serbia are explored to identify common underlying causes using a novel taxonomy of contributing factors across aspects and health system levels. The GDPR has an extraterritorial application for the non-EU data controllers who process the data of EU citizens and residents, which mainly affects private practices used by medical tourists from the EU, public health care institutions frequented by foreigners, as well as expatriates, dual citizens, tourists, and other visitors. Serbia generally does not have well-established procedures to support international research collaborations around its health data. For smaller projects, contractual arrangements can be made with health data providers and their ethics committees. Even then, organizations that have not previously participated in similar ventures may require approval or support from health authorities. Extensive studies that involve multisite data typically require the support of central health system institutions and relevant research data aggregators or electronic health record vendors. The lack of a framework for preparation, anonymization, and assurance of privacy preservation forces researchers to rely heavily on local expertise and support. Given the current limitation and potential issues with the legislation, it remains to be seen whether the move toward the GDPR will be beneficial for the Serbian health system, medical research, protection of personal data and privacy rights, and research capacity. Although significant progress has been made so far, a strategic approach is needed at the national level to address insufficient resources in the area of data protection and develop the personal data protection environment further. This will also require a targeted educational effort among health workers and decision makers, aiming to improve awareness and develop skills and knowledge necessary for the workforce.

## Introduction

### Background

The European General Data Protection Regulation (GDPR) 2016/679 [1] was established in April 2016, replacing the Data Protection Directive 95/46/EC and detailing the constraints around the processing of individuals’ personal data inside the European Economic Area. As of May 2018, all relevant institutions in the member countries have to comply with the GDPR or face significant fines. This regulation also has a notable effect on the European Union (EU) candidate countries, which are undergoing the process of harmonizing their legislature with the EU, as part of the accession process. The GDPR requirements also have a strong global impact, necessitating technological advances in data collection, sharing, and analysis and increasing economic interest in health data, thus bringing forward the need for new data-sharing policy frameworks [2].

The Republic of Serbia is an example of a country, which is not a member of the EU but where the GDPR is highly relevant. Serbia is moving toward full GDPR alignment through the new 2018 Personal Data Protection Act (PDPA18), which contains the majority (though not all) of the provisions of the GDPR, creating a specific regulatory environment in Serbia’s interactions with other EU countries, including its immediate neighbors. Given the duration of the EU accession process in Serbia and other candidate countries, namely, Northern Macedonia, Albania, Montenegro, and Turkey, this situation may continue for a prolonged period. 

### Objectives

This paper uses Serbia as an example to highlight the issues in the implementation of GDPR-aligned legislature in an EU candidate country and provides guidelines for any future adopters. As one of the very few low- and middle-income countries (LMICs) in Europe, Serbia is increasingly seen as an attractive ecosystem for LMIC implementation research projects, and this paper provides some recommendations for conducting such research in the local setting.

## Serbian Privacy Protection Landscape

The 2013 Patients' Rights Act, amended in 2019, explicitly stipulates that (1) all health workers and their associates shall safeguard the confidentiality of personal and health data; (2) particularly, sensitive data must be handled in a way that always ensures privacy and confidentiality; and (3) all health care institutions and other legal entities handling such data are obliged to establish and maintain appropriate security systems and measures. This act explicitly obliges the health care workers and others who process these data to preserve confidentiality unless consented by the patient or legal representative in writing or by a court decision.

The original 2008 Personal Data Protection Act (PDPA08) introduced the role of the Commissioner for Information of Public Importance and Personal Data Protection, who was put in charge of implementation monitoring and enforcement of the act. Numerous cases of data breaches or misuse of personal and health data have been reported, resulting in a series of relevant recommendations, warnings, and decisions [3]. The Commissioner’s interventions generally involved not only corrective actions but also fines and court filings, for example, the first fine for the unauthorized processing of personal data was for the illicit processing of health data. However, criminal convictions and sanctions by professional bodies, for example, the Medical Chamber of Serbia, have been rare. In 2018, 1452 data protection–related inspections were completed; in 956 cases, the warning or decision was followed; 16 cases produced requests for the initiation of misdemeanor proceedings; and in 6 cases, criminal complaints were filed [4]. Of 1450 initiated inspections, 63 were in health care organizations. From 2010 to 2018, the Commissioner submitted 39 criminal charges, which led to 2 prosecutions, resulting in one 6-month probation and one acquittal. The procedure for 18 criminal charges was still ongoing at the end of 2018. The situation with misdemeanor proceedings is far more favorable; during 2018, the Commissioner filed 19 requests and received 23 decisions of the misdemeanor courts, of which 18 were convictions. The sentences imposed have all been at the mandatory minimum.

As a comparison, the Personal Data Protection Agency in Bosnia and Herzegovina received 148 complaints and conducted 40 *ex officio* proceedings in 2018; of these, in 5 cases, measures related to health care institutions and health insurance were adopted, with two more measures in cases related to health data [5].

The main types of recurring incidents addressed by the Commissioner Office are as follows:

1. Incidents related to health documents in the form of paper and information visible in the premises of health care organizations [6,7]: In these cases, which formed the majority of reported incidents, the documents containing patients’ health status were kept at health premises in an unsafe manner or were even made available to visitors. In one instance, the information about the patient’s HIV status was attached to their bed [8].
2. Improper disposal and even reuse of paper with personal or medical data [9].
3. Improper disclosure of information on the health status of celebrities without proper consent [10,11].
4. Personal health data and records being leaked to the media to humiliate individuals for political purposes [12,13].
5. The case of the central Integrated Health Information System (IHIS) implemented by the Ministry of Health (MoH): Between 2016 and 2018, the Commissioner issued many opinions, warnings, recommendations, and conclusions on several technical and legal issues, such as serious failures in the protection of personal data that involved a high risk of unauthorized access and other large-scale rights abuse [14]. Most of these issues were resolved by 2018 [15], but the policy documents related to IHIS and handling of the data contained in it were not made public.
6. Misuse of health data for commercial and marketing purposes [16,17].
7. A patient mobile app for access to IHIS included direct marketing and profiling by the vendor in its terms of use and privacy policy [18]. There is no indication that such uses of the data from IHIS have occurred, but the terms of services for this app are currently empty and are missing for the corresponding Web app [19].
8. Passing information between different government institutions (police collected mental health diagnoses of people in some municipalities, in line with outdated regulations; they subsequently deleted them) [20].
9. Resolving contradictions in the law on whether police can collect health data about suspects and victims of crime or if such data can only be issued with a court warrant or with the authorization of the individual in question [21].
10. Unauthorized, excessive, or disproportionate collection of data within the health system. Some local National Health Insurance Fund (NHIF) offices collected medical documents and then deleted them, prompting the NHIF director to ban such practices [22,23].
11. A student health center collected data on students’ sexual orientation without prior authorization during regular health checkups; the data had to be deleted when the Commissioner intervened [24].

### Contributing Factors

The mentioned incident types were analyzed to identify the underlying causes (Table 1). Owing to a lack of suitable taxonomies for data protection–related incidents or behaviors in the health sector, a working classification based on existing taxonomies for telemedicine [25] and electronic health [26] is outlined to provide insight into possible causes and deficiencies. Classifications from two areas that are significantly dependent on trust, chronic obstructive pulmonary disease self-management [27] and shared decision-making [28], provided a blueprint that was further refined by observing four health system levels (patient, practitioner, organization, and system) and five aspects (attitude, information and communication, skills and tools, resources, and context). The resulting classification is given in Textbox 1.

**Table 1:** Factors causing (numbered) types of data management problems and breaches in Serbia (columns represent health system levels, and rows denote aspects).

Aspect	Health system level			
	Patient	Practitioner	Organization	System
Attitude	1, 11	1-3, 6, 11	1-6, 8, 10, 11	1, 3-6, 8-11
Information and communication	1, 11	1-3, 6, 11	1, 3, 5, 6, 8-11	1-6, 8-11
Skills and tools	1, 11	1, 6	1-4, 6, 8, 9, 11	1-11
Resources	N/A^a^	1, 2, 6, 11	1, 2, 4-6, 8-11	1-11
Context	1, 11	1-3, 6, 11	1-6, 8-11	1, 3-5, 7-9, 11

^a^N/A: not applicable.

The impact of the factors (Textbox 1) was considered for the incident types listed. Individual case types and factors were associated only if it was concluded that a change in the factor could prevent the related privacy or security events from occurring. The resulting impact matrix is given in Table 1. Opaque and cumulative relationships between factors and situations were not considered. For example, it would be beyond the scope of this paper to consider whether a change in attitude or skills of a large group of affected patients would result in a change in the orientation and priorities of health care providers or system-level decision makers.

The indicative associations can be used to draw some high-level conclusions, even without performing a full quantitative analysis of incidents. Distribution of data management problems across health system levels is broadly identical for all aspects (Table 1) and uniform at each level in terms of aspects (ranging from 23/122, 18.9% to 25/122, 20.5%). Although patient-related factors could affect the outcome in just a few situations (8/122, 6.6%), the impact of factors related to practitioners (21/122, 17.2%), health care organizations (44/122, 36.1%), and health system (49/122, 40.2%) is considerably greater. The impact of authority is even more evident if it is, for each situation type, checked whether the contributing factors at one level are matched with contributing factors of the same aspect at adjacent levels. Looking toward the level above, this occurs in 92% (67/73) of cases: the presence of an aspect is almost always matched by a corresponding aspect at the level immediately above. In the opposite direction, this correlation is only 58.8% (67/114). In other words, the contributing factors tend to chain up all the way to the system level.

Many health care organizations in Serbia do not have internal acts regulating data protection; some regulate the protection of personal data in their statutes or business ethics codes [29]. Although health professionals may have basic training in the use of their information technology (IT) systems, they are typically not trained in ethical awareness and protecting sensitive patient data. Most commonly, the protection and privacy rules related to the use of electronic health records (EHRs) are introduced upon vendors’ initiatives and with the involvement of health care organizations’ managers, or they are established after an incident or the Commissioner’s intervention.

**Factors that hinder or support data protection.**

*Patient-level factors*

- Attitude: motivation, awareness, and trust in practitioners and system
- Information and communication: understanding and knowledge of rights, risks, roles of subjects, and pros and cons of implementing data sharing and privacy
- Skills and tools: the ability to control one’s health data and skills needed to act
- Resources: social and support networks
- Context: personal circumstances, socioeconomic context, and emotional and cognitive status

*Practitioner-level factors*

- Attitude: awareness, sensitivity, accountability, focus on patients, trust in the system, and openness to change
- Information and communication: understanding and knowledge of norms, practices, and data usage by the system
- Skills and tools: use of data and communication tools
- Resources: access to multidisciplinary support team and time for reflection
- Context: personal circumstances, fatigue, frustration, or resignation and professional habits

*Organizational factors*

- Attitude: organizational culture; managerial leadership, encouragement, and feedback; and organizational responsibility
- Information and communication: teamwork, effective communication, and coordination
- Skills and tools: procedures, workflows, and data management tools
- Resources: management competence and capacity and allocated time, staff, and other resources
- Context: priority relative to other aspects of care delivery, standard operating procedures, and management vulnerability

*System-level factors*

- Attitude: culture of health care delivery; leadership, encouragement, and feedback; and strategic orientation toward patient and population outcomes
- Information and communication: communicated values; education, materials, campaigns, and support for all levels
- Skills and tools: managed policies, legislation, standards, and guidelines; accreditation and certification criteria for health care organizations and information and communication technology vendors; professional education and licensing; sanctions; monitoring and reporting capabilities and instruments; consistency promotion and support
- Resources: governance capacity and competence and capacities of data protection and health system supervisory authorities
- Context: externally managed policies, legislation, standards, and guidelines; market; binding arrangements; and international alignment and harmonization

The Serbian IHIS is no exception to health data centralization initiatives in other countries, which have also faced controversies related to legal complications; project and data management; and communication, expectations management, and public perception [30].

It is noteworthy that, so far, there have been no reports of any large personal data leaks from the health system, despite a number of such breaches in other domains in Serbia, for example, the unauthorized release of personal data of more than 5 million citizens on the website of the Privatization Agency in 2014, which resulted in no convictions [31].

The NHIF has been an exception to this situation for years. After every Commissioner’s intervention, it promptly defined the corresponding privacy-related policies and codes of conduct and provided detailed answers to all requests and questions related to data protection [32]. Its employees are required to sign confidentiality agreements [29]. The NHIF has been establishing the capacity in this field along with the development of its infrastructure, systems, and services.

Given the highly centralized approach toward data protection imposed by the PDPA08, the Commissioner’s work has made a great impact on the attitude toward health data and the protection of personal data, in general, in Serbia. However, a lack of resources prevented the Commissioner from acting within their full capacity. It has been claimed that the Commissioner, with the available capacities, could not fully fulfill their mandate [4,29].

### Interaction With European Union Countries

Owing to the close political, social, and economic links between the Balkan countries, some of which are full EU members, the GDPR also greatly impacts Serbian health care organizations in their everyday operations.

Along the borders with Serbia’s EU neighbors—Croatia, Hungary, Romania, and Bulgaria—many people have dual citizenship. There is a growing number of EU citizens who establish residence in Serbia, as it grows closer to the EU and becomes more attractive for living. More importantly, there are municipalities in Serbia with a significant population of expats who, after being granted EU residence permits or citizenships and ending the job in a new country, decide to spend a significant portion of their time back in Serbia. All such individuals are likely to receive regular primary care, specialist services, and perhaps even long-term care from public health care organizations. Incidentally or not, many municipalities with returning expats are located in South-Eastern Serbia along the Pan-European Transport Corridor X, which also brings some occasional patients. Health care organizations at such locations, similar to other organizations that regularly work with EU citizens, should assess the influx of EU citizens, become fully GDPR complaint, and have a data protection officer (DPO) and EU representative [33].

At the time of writing this paper, there is only one international health care organization operating in Serbia that is in a position to use its international data protection and GDPR expertise on the local market [34,35]. In addition, any larger local clinics and provider associations that target customers from the EU had to make preparations for GDPR compliance well in advance [36,37].

### Implementation Challenges

Companies focused on the local market also need to align with the GDPR because of the changes in the Serbian law. However, this will be difficult even for the large entities in the public sector. Most of them will not be incentivized to establish a GDPR-compliant program, assess the current level of compliance, audit all personal data processed, and review their data protection policies. Many entities may also assume that the rules imposed by the PDPA18 are sufficient and will be unaware of the GDPR requirement to have an EU representative if they have *nonoccasional* EU patients. Other GDPR requirements, such as maintaining data processing records, establishing breach procedures, nominating DPOs, or conducting privacy impact assessments where needed, are all covered by the PDPA18. As the DPO’s role often overlaps with existing executive functions, although data protection may go against other business objectives [38], these officers may, in addition to their internal mandate, rely on an external authority to fulfill their duties and lead organizations toward the new rules imposed by the law, which will inevitably have an impact on the current work process, comfort, and previously set goals.

## Responsibilities of Data Protection Officers

Engaging a dedicated person to deal specifically with personal data will be increasingly difficult in the ongoing austerity situation where Serbian public health care organizations are pressured by the MoH and NHIF to reduce the nonmedical staff. This reflects the overall situation in Serbia, where many companies have no one to deal with personal data and its protection and where, in large systems, services are decentralized with some data stored on paper and some on company servers [39]. Public health care organizations will most likely try to transfer these responsibilities to the MoH or to extend their service contracts with the IT vendors and support contractors. Although central authorities and external contractors can be of help, it is ultimately the health service providers who need to take responsibility. One of the first things they must do is to improve their understanding of the data categories they process, invest in the right kind of technology to secure the information, and implement appropriate technical and organizational measures for data protection. With that in mind, the new DPOs will likely be primarily recruited from the current managerial staff, despite the need for specific skills and full-time engagement.

At the health care–provider level, similar issues were reported in some EU countries, where the GDPR transition process was described as slow and accompanied with insufficient training, problems in the nomination of DPOs, and a lack of awareness of fines [40].

At the national level, the new regulatory role of the Commissioner is about to change. Instead of being in charge of maintaining the registry of personal data collections, dealing with complaints, and, often, acting as the ruling and fining authority, its focus will shift toward support, interpretation, and overseeing reported breaches, as has been the case in the countries that have adopted the GDPR [38]. It will also more frequently assume the role of an involved party in court proceedings on data protection. In EU countries, on the introduction of GDPR, the national regulators were initially overwhelmed with 72-hour breach reports and requests for guidance on the GDPR [38]. As the Commissioner has been reportedly understaffed even to carry out the old legislation [4,29], it is reasonable to assume that they will face similar challenges again, particularly during the initial period of the PDPA18 implementation.

## Transition to General Data Protection Regulation

The new PDPA, adopted in 2018, came into force in August 2019, replacing the PDPA08. The PDPA18 abolished the Central Personal Data Register, as the responsibility for keeping records of processing activities was fully transferred to data controllers. During the transition period, the controllers continued to have the obligation to submit the records on data processing to the Commissioner and to notify them on their intent to establish data processing, despite the abolition of the central register. In addition, although the PDPA08 required data processing to be based on either personal consent or some legal act mandating the processing of specific data content, the PDPA18 defines the lawfulness of processing in the same way as the GDPR.

As part of the wider process of harmonizing Serbia’s legislature with the EU, the PDPA18 has been modeled after the GDPR and is largely compliant with it. Conversely, its territorial application is extended to the processing of personal data of those domiciled or residing in Serbia if the controller or processor is based in Serbia, or the processing is related to the provision of goods or services in Serbia, or data subject monitoring performed in Serbia, regardless of where the data processing is carried out. The PDPA18 introduces a more precise definition of personal data as well as the protection mechanisms and rights for individuals that correspond to those provided by the GDPR. It introduces the same technical and organizational personal data protection measures, the personal DPO role, the privacy impact assessment, and breach procedures. Finally, it regulates the transfer of personal data out of the country, following EU procedures for determining whether the destination country can ensure an adequate level of data protection.

Although the PDPA18 doubles the maximum penalty provisions compared with the PDPA08, bringing them into 50,000 to 2 million Serbian Dinar range (US $461.65 to US $18,464.32), these are still smaller than the penalties imposed by the GDPR, which may reach higher than 20 million € (US $2.2 million) or 4% of the global annual turnover. As a comparison, the fines that can be incurred to public authorities in Romania range from 2000 to 43,00,000 € (US $2280 to US $45,500) [41], which may yet be investigated by the European Commission as too low and discriminatory for other organizations [42]. This relatively low fine level may negatively impact the effective implementation of the PDPA18, in addition to all organizational, governance, juridical, and other challenges observed during the application of the PDPA08.

During the first year of GDPR in the EU, application fines have been imposed on several large corporations [38], with disproportionately fewer cases raised against small and medium–sized enterprises and health care organizations because of limitations in national regulators’ capacity. A similar situation may be expected in the initial stages of the PDPA18 application in Serbia. However, dealing with health and health care data is not only finable by both the PDPA and the Patients’ Rights Act but is also a criminal act punishable by up to 3 years in prison. Furthermore, while previously the Commissioner could issue fines, this responsibility now lies with courts, which have so far largely been issuing minimal fines, as described above.

Another controversial change is related to privacy restrictions stipulated in Article 23 of the GDPR. The corresponding article of the PDPA18, when it was publicly discussed at the end of 2017, stipulated that the related citizens’ rights and data protection obligations could be restricted by law only. In the adopted Act, *by law only* was omitted. The PDPA18 literally copies from the GDPR the reasons such as national and public security, defense, dealing with criminal offenses, or important objectives of general public interest, but the second paragraph of the article does not mention that the corresponding legislative measures shall contain specific narrowing provisions. Instead, it turns the required provisions into elements that must be taken into account, as appropriate, at the point of restriction of rights and obligations. Many people fear that this, accompanied by weak checks and balances, leaves room for the authorities and even companies to handle personal data in a way that would undermine citizens’ rights.

### Personal Data Protection Act, 2018: First Implementation Experiences

The PDPA18 does not prescribe any specific conditions for DPOs in terms of education, expertise, skills, and experience in the field. Although PDPA18 replicates the parts of the EU Data Protection Directive 2016/680 [43] that complement the GDPR concerning the position of the DPO, Serbian DPOs are not supported with guidance and clarifications as provided in the EU guidelines [44]. To help organizations and DPOs, the Commissioner created a brief guide [45].

However, to perform their function, DPOs need to possess diverse and highly heterogeneous knowledge and relevant work experience. The nature of the work also requires DPOs to be at a part of top-level management. Some large organizations may be able to identify suitable individuals among the top rank and assign them the DPO role in an addition to their related duties, but this is not the case with the public health sector in Serbia, where the members of the management originate from health care professions. At the same time, the current austerity directives prohibit the employment of nonmedical staff.

In recognition of this situation, the Commissioner requested a 1-year deferral of the PDPA18 to September 2020 [46] to allow for additional time to build the capacity and raise awareness, and to allow the investments in IT and data security to bear fruit. In addition, the Commissioner has not been provided with the financial resources necessary for their new competences. Although the Serbian Commissioner, also in charge of information of public importance, has 78 employees [4], the Romanian Data Protection Authority has grown from 50 to 85 employees to be able to oversee the GDPR implementation [41].

One week after the beginning of the PDPA18 application, out of tens of thousands of controllers, only 192 registered their DPOs with the Commissioner [47]. It is yet to be seen how these organizations will deliver operational procedures and processes required or implied by the PDPA18. An analogous example can be found in Portugal, where of 57 surveyed clinics, 4 reported to be in compliance with the GDPR, but only 1 had actually designated a DPO [40].

Although the lack of information and skills in the GDPR countries was compensated by private sector companies, which started providing training, materials, legal consultancy, and certification, and even outsourcing DPOs, such services were launched in Serbia only after the PDPA18 application had been started in August 2019.

## Discussion

### Data Protection Enforcement in Serbia

Data protection culture in Serbia is relatively new and has been influenced by the PDPA08 and the work of the Commissioner. Now that GDPR alignment is in progress, past experiences are worthy of further consideration. The contributing factors that have been at work over the past decade are still of great influence. Moving to the PDPA18 emphasizes the roles of DPO, health care organizations’ management, and the courts. It is particularly worth to look back at the history of court verdicts so far. Although all past health data breaches were relatively small, none were processed as criminal offenses. This could be attributed not only to Serbian courts’ lenient policy in data protection matters but also to the reasoning that it is better to raise awareness and change privacy culture by dealing with incidents through inspection and public warnings than to doom the Commissioner’s mission by losing a few high-stake cases or triggering a coordinated political backlash. Given the decentralized approach of the GDPR and PDPA18, the course of data protection and related practices in Serbia will be increasingly affected by the attitude and capacity of courts and health care organizations.

### Research Using Serbian Health Data

The use of cross-institutional data for scientific research in Serbia is currently limited. There are only two exceptions. One is public health and system–level data collection, as there are mechanisms in place that are used for population health surveillance by the Institute of Public Health as well as those established by the NHIF and MoH to track and monitor individual service provision and overall performance of the health care system. The other exception is data collected in clinical trials. Unfortunately, both have specific primary purposes and do not support flexible cross-institutional or posterior arrangements that would facilitate scientific research.

Except for clinical trials, the current legislation does not regulate the conditions for health data reuse in scientific research. Most health care organizations have ethics committees that monitor and analyze the application of ethical standards in the delivery of health services, approve and oversee clinical trials and scientific research, and manage the evaluation and introduction of new health technologies. However, their standard operating procedures are primarily tailored for clinical trials. It is, therefore, difficult to establish other types of research or multitier data collaborations unless they are conducted under the direct auspices of central institutions of the health system and rely on the data these institutions already aggregate regularly.

By following the GDPR, the new legislation details for the first time the application of pseudonymization and encryption of personal data in the processing of personal data. This also clarifies when data subjects need to be informed, exceptions in rights and purpose, and limitations concerning storage for scientific or historical research and statistics.

This partially bridges the gap between Serbian legislation and the needs of the research community. To further support health data research, still missing is a specific regulatory framework and codes of conduct in this area, including supervisory and advisory bodies that would safeguard data sharing, linkage, and use in scientific research. An impartial mechanism would ensure adequate pseudonymization, anonymization, and sufficient-level aggregation of used health data or linked health and other personal data from various sources, thereby preventing reidentification of individuals by linking with other available information. Such an entity could potentially be established within the National Open Data Initiative portal [48], which promotes the use of open data in sectors such as security, education, energy, governance, health, and environment. It provides access to datasets and an app program interface for data browsing, download, publication, and updating.

Except in the domain of clinical trials, as detailed above, Serbia does not currently have well-established procedures to support international research collaborations around data created in Serbian health care organizations.

In minor ventures, arrangements can be made with organizations’ management bodies and their ethics committees and then secured through contracts. Even then, small organizations that have not previously participated in similar ventures may require approval or support from health authorities. The operational aspects of data collection and processing could be addressed either by providing them with a custom data entry tool or by using the existing EHR system to get the historical data and to collect additional information. The latter approach typically requires the involvement of the EHR vendor, which can also anonymize or pseudonymize the data before they are handed over to researchers.

Extensive studies that involve multisite data typically require the support of central health system institutions, such as the MoH, NHIF, or the National Institute of Public Health, as well as any relevant research data aggregators and EHR vendors.

Owing to the lack of a framework for preparation, anonymization, and assurance of privacy preservation, researchers must rely heavily on local expertise and support.

### Direct Impact of General Data Protection Regulation on Health Care

Serbia is a popular destination for medical tourism because of low prices, quality services, and geographical proximity [49]. The most popular specialties include dentistry and minimally invasive plastic and urogenital surgery, with gender reassignment being one of the areas where Serbia is particularly prominent [50]. There are also regular tourists from the EU, business visitors, and those in transit to and from member countries such as Greece, Bulgaria, and Turkey, which, similar to Serbia, are a country of origin for many EU citizens and residents.

The GDPR has an extraterritorial application for the non-EU data controllers who process the data of EU citizens and residents. This primarily affects Serbian private practices targeting EU citizens, although some visitors end up in public health care organizations.

At the time of collecting their data, EU patients must be informed clearly about many things, including which data are being collected, which organizations will see the data, and the use data will be put to. Although health care providers may rely on the explicit consent or contract to establish a lawful base for data processing, they also must make sure that all conditions and rights imposed by the GDPR are satisfied, while the ways they are implemented are practical and achievable with the patients. A particular challenge in this is to ensure adherence to the local legal reporting and audit obligations while staying within the expectations and comfort zone of international patients.

In addition to the standard GDPR requirements for EU entities, a company that is without an office in one of the EU member states but still providing products or services in the EU or systematically monitoring or collecting the data on the people from the EU must appoint a legal representative who is residing in the EU. Such a representative person or company is the main contact for any questions and concerns regarding data protection from any EU citizen or supervisory data protection authority. The only exception to the obligation of having a representative is if the processing of personal data only happens occasionally and is, therefore, unlikely to result in a risk to the rights and freedoms of natural persons. The term *occasionally* is ambiguous in this context. Although it is likely intended to refer to incidental patients visiting Serbia for nonmedical reasons or people in transit who are most likely to be injured in traffic accidents, should it also apply to people coming to Serbia to receive medical services? As such decisions are probably made based on information and marketing materials available in the EU and the service is offered in the EU, the service provider should establish an EU representative.

### Impact on Relationships With European Union Countries

Owing to potentially huge GDPR penalties, the EU insurers and other companies in the health sector may decide not to cooperate with Serbian entities that do not comply with the regulation. Accordingly, health care organizations in Serbia must decide whether the cost of implementing the regulation is offset by the potential value of medical tourism from the EU. For small companies that are not directly soliciting business in the EU, the risk of becoming an enforcement target is small but still real, as such companies are currently most likely not to be fully GDPR compliant. Fortunately, the PDPA18 already requires compliance with most of the GDPR, except toward EU citizens and residents and concerning the EU representatives. This makes it much easier to comply with the GDPR once nationally mandated requirements are met.

The same applies to the additional requirements imposed by the individual EU member states, as the GDPR allows individual EU states to adopt separate rules that can be tougher than the basic GDPR norms. As far as Germany, the country of residence for many Serbian expatriates and a major economic partner, is concerned, the most relevant regulatory information for Serbia is the specifics of the German Federal Data Protection Act (Bundesdatenschutzgesetz). It has stricter rules on DPOs and defines damages that are not readily quantified in money, such as compensation for pain and suffering [51]. Even if these liabilities are not directly applicable to Serbian health care service providers, they may create substantial economic risks through German partners, such as insurers or providers of intermediary services.

One could argue that the safest short-term strategy for a health care provider in Serbia is to pass on all recorded health data to the foreign patient once the episode of care is over while keeping financial records that are required by law. This would reduce the long-term risks and emphasize the notion of *occasional*. However, such providers would still be processing sensitive personal data, and this would conflict with their standard operating procedures and local legislation. Finally, once Serbia joins the EU, such a practice would be against the EU Directive 2011/24 on Patients’ Rights in Cross-Border Health Care, the Regulation 910/2014 on Electronic Identification and Trust Services for Electronic Transactions in the Internal Market, and whatever comes as the follow-up of the European Commission Recommendation 2019/243 on a European EHR exchange format. The same cross-border interoperability mechanisms will have to be provided for Serbian citizens traveling abroad so that doctors from other EU countries can access their health records (and vice versa).

### Storing Personal Data on Cloud Platforms

A shift toward the GDPR may have an unexpected side effect. In the legal system of the Republic of Serbia, there are no specific provisions regulating cloud computing services. Given the prescriptive nature of PDPA08 and sectoral laws related to health data, organizations were reluctant to adopt the software-as-a-service model and put their data on the cloud or hand them over to external service providers. This resulted in local IT deployments that created maintenance issues for the organizations and the vendors working with those organizations. The PDPA18 and the GDPR put a different angle on the relationship of data controllers and processors and often dogmatically debated issues of data ownership and stewardship. The PDPA18 has the potential to facilitate the adoption of novel technical solutions; however, organizations do require practical guidance, particularly for small health service providers that typically do not have the resources and expertise to develop related policies and procedures, establish partnerships, and lead on implementation.

### Conclusions

Although Western European countries adopted their first laws on data protection during the 1970s, Serbia introduced the initial regulation in the area more than three decades later. Over the past 10 years, significant efforts have been made to compensate for this lag, culminating in the recent adoption of an act that is largely in line with the GDPR. The PDPA18 is radically changing the existing approach to data protection through the decentralization and sharing of responsibilities. However, Serbia, similar to Romania, the United Kingdom, and Spain [42], made a number of problematic derogations in its GDPR-implementing legislation, which will need to be addressed during the EU accession process to raise the standard of data protection to an acceptable level.

The examples presented indicate that, in addition to the law, it is necessary to change the culture of data governance and introduce many systemic improvements. The established regulation, the work of the Commissioner, the extensive coverage of the topic by the media, and the growing awareness of individuals about the importance of personal information protection have all contributed to a significant improvement in Serbian data protection landscape.

The fines in the PDPA18 are relatively minor, particularly for large organizations. In addition, some organizations are concerned with whether they can meet all the requirements of the GDPR and may decide to risk the fines instead. More importantly, health care organizations at all levels lack the necessary regulatory and sectoral governance capacity to supervise the transition, enforce the rules, and provide the needed support and assistance.

Serbia has embraced a comprehensive approach toward data protection introduced by the GDPR. This is in contrast to the vertical-limited approach of the US Health Insurance Portability and Accountability Act rules, which provide stronger sectoral downstream protection for health care providers and patients but lack sufficient upstream controls toward *big data* brokers [52]. With the Commissioner having a central role, the elements of cross-sectoral perspective were already introduced by the PDPA08. However, the vertically focused governance is likely to be adopted in the Serbian health sector, and the risks associated with sectoral enforcement and potential reduction in the influence of regulators, which was perceived as a potential threat in the United States [52].

Given the current limitation of its health and data governance systems and potential issues with the forthcoming legislation, it remains to be seen whether the move toward the GDPR will be beneficial for the Serbian health system and medical research in terms of the protection of personal data and privacy rights and research capacity. Although significant progress has been made so far, direct application of implementation methods designed for more advanced health data environments can be risky, but they could also stimulate the community to move forward.

Serbia needs a strategic approach at the national level, systematic elimination of problems arising from insufficient resources in the area of data protection, and further development of a modern personal data protection regulatory and institutional environment. This can only be achieved through a targeted educational effort among health workers and decision makers, aiming to improve awareness and develop the necessary skills and knowledge in the workforce.

Finally, to facilitate health data research projects on a large scale, a decentralized approach to data protection governance is needed, together with new bodies responsible for the development of policies and guidelines, and design and monitoring of improvement activities, possibly with a separate mandate dedicated to health care. It is particularly critical to design instruments that would stimulate and support institution managers and health care professionals in enhancing privacy and data protection. Only such an approach will ensure long-term sustainability and progress in this area.

## Abbreviations

DPO: data protection officer
EHR: electronic health record
EU: European Union
GDPR: General Data Protection Regulation
IHIS: Integrated Health Information System
IT: information technology
LMIC: low- and middle-income country
MoH: Ministry of Health
NHIF: National Health Insurance Fund
PDPA08: Personal Data Protection Act, 2008
PDPA18: Personal Data Protection Act, 2018
